# Relationship between Quality of Life and Adult Attachment Factors in Mothers of Children with and without Type 1 Diabetes

**DOI:** 10.3390/ijerph20237109

**Published:** 2023-11-25

**Authors:** Maria de Nazareth de Lima Carneiro, Daniela Lopes Gomes, Arthur Andrade da Fonseca, Rachel Coelho Ripardo

**Affiliations:** Nucleus of Behavior Theory Research, Federal University of Pará, Belém 66087-110, Brazil; danielagomes@ufpa.br (D.L.G.); arthur.fonseca@ics.ufpa.br (A.A.d.F.); rcripardo@ufpa.br (R.C.R.)

**Keywords:** quality of life, adult attachment style, maternity, type 1 diabetes mellitus

## Abstract

The mothers of children with a specific clinical situation such as type 1 diabetes mellitus may have a higher level of stress, causing a worse perception of their quality of life, greater anxiety, and greater avoidance (adult attachment factors). The objective of this research was to verify if there is a relationship between the adult attachment factors of mothers of children with and without type 1 diabetes mellitus and the perception of the quality of life of these mothers. This survey was carried out from July to September 2022, with mothers of children aged 5 to 10 years, with and without diabetes. The data were collected through an online questionnaire, with socioeconomic data from the attachment scale Experience in Close Relationship—(Reduced), and the questionnaire on the quality of life, the WHOQOL-abbreviated questionnaire. For statistical analysis, the Statistical Package for Social Science 24 was used. A total of 45 mothers of children with DM1 and 55 mothers of healthy children were evaluated. The mothers of children with DM1 had a worse perception of their quality of life when compared to the mothers of healthy children (*p* < 0.05), with no difference in terms of the attachment style. Therefore, it is understood that actions aimed at improving the quality of life of these mothers are necessary.

## 1. Introduction

In the first months and years of life, according to attachment theory, the child/infant and their main caregiver create a bond that represents physical and emotional security in situations of discomfort [1]. This depends on the first lived experiences and is named the attachment style, which is classified into secure and insecure, and it presents anxiety and avoidance factors, thus, if the individual manifests high anxiety and/or avoidance factors, their attachment style will be classified as insecure [2].

Such characteristics will be reflected in relationships throughout the individual’s life in rewarding or exhausting situations, such as motherhood [3,4]. Mothers usually go through situations of pleasure and stress [5,6], but when the child presents with a specific clinical situation, such as type 1 diabetes mellitus (DM1), the literature points to higher levels of maternal stress, due to the need for glycemic control, restrictions, and other care related to the child. Because of these issues, many of these mothers call themselves “pancreas mothers” [7,8,9]. This may be associated with the perception of a worsening of the mother’s quality of life, which would be directly related to physical health, psychological state, social relationships, relationship with significant aspects of the environment, and with greater anxiety and attachment avoidance as an adult [1,10]. For these mothers, being the main caregiver generates meanings that are often not observed by the health team, whose attention is commonly focused on childcare, forgetting that the relationship established between the child and the mother, in the care process, intervenes directly in the health condition of the child [11,12].

Much progress has been made in attachment theory in different approaches, such as attachment in adolescents and its interference in adulthood [13], adult attachment and marital bonds [14,15], attachment and its influence on paternal involvement [16,17,18], and attachment and mourning [19], among others.

There are few studies that relate to chronic diseases and attachment style. In a study with patients diagnosed with type 1 and 2 diabetes over the age of eighteen undergoing treatment for two years, it was found that their insecure attachment style would show as a lower adherence to treatment and consequently higher levels of glycated hemoglobin, which means that apparently the attachment style could also have some relationship with the treatment of these patients [20].

However, so far in the literature, no studies have been found that examine the relationship between adult attachment and the perception of quality of life in mothers. Therefore, with the following hypothesis: “There is an association between the attachment insecurity of mothers of children with type 1 diabetes with the worst perception of quality of life of these mothers”, this research aims to verify whether there is a relationship between the adult attachment factors of mothers of children with and without type 1 diabetes mellitus with the perception of quality of life of these mothers.

## 2. Materials and Methods

### 2.1. Type of Study

This study is cross-sectional, descriptive, and analytical, carried out with a convenience sample. Data were collected from July to September 2022, and the collection was performed using an online form, built on the Google Forms^®^ platform. Participants were recruited through the researchers’ Instagram^®^, Facebook^®^, Telegram^®^, and WhatsApp^®^ social networks and specific diabetes-themed social networks.

### 2.2. Participants and Inclusion and Exclusion Criteria

A total of 136 adult mothers from all over Brazil participated in this research. Inclusion and exclusion criteria were determined in two steps.

In the first stage, the mothers who were not the main caregivers of their children and the mothers of children aged less than 5 years and greater than 10 years were excluded. After applying these criteria, 26 mothers were excluded, as 9 did not live with their children, 11 had children younger or older than the established age group, and 6 maintained shared custody of their children, that is, they spent only 15 days each month with them. This left a total of 110 mothers who met the inclusion criteria, such as being the main caregiver of their children, residing in Brazil, and agreeing to participate in an online survey.

These mothers were divided into two groups: group “A”, composed of mothers of children with DM1, with a total of 53 participants, and group “B”, with mothers of children without chronic diseases, with a total of 57 participants.

After this division, the second stage of the inclusion and exclusion criteria was carried out, which referred to issues of the child’s illness. For group A, the child could not have another chronic disease besides DM1, and in group B, the child could not have no chronic disease.

After applying these criteria, 8 mothers from group A were excluded, leaving a total of 45 mothers, and in group B, 2 mothers were excluded, leaving a total of 55 mothers.

### 2.3. Research Instruments

As instruments, a sociodemographic and clinical questionnaire were used; the Experience in Close Relationship—Reduced scale (ECR-R-Brazil) [21]; and the WHOQOL-abbreviated questionnaire [22].

(a) The sociodemographic and clinical questionnaire prepared for this study, in which items such as age, marital status, education level, average family income, among others, such as height and weight were collected, were used. Through these last variables, it was possible to verify the Body Mass Index (BMI), recognized as an international standard to assess the proportion of overweight and obese individuals in populations [23]. It is calculated by dividing weight (kg) by height squared (meters).
(b) The Experience in Close Relationship Scale—Reduced (ECR-R-Brazil) contains 10 items, through which anxious and avoidant attachment factors are measured. Its result is expressed as an arithmetic mean, with the result for the anxiety factor being calculated by the average of even items (2, 4, 6, 8, and 10), and the result for the avoidance factor obtained through inverting the items 1, 3, 5, and 7, and the calculation of the arithmetic mean of the odd items. The higher the anxiety and avoidance scores, the more insecure the respondents will be considered in terms of their attachment style. If the respondent has low anxiety and avoidance factors defined by this scale, she will be considered as having a secure attachment style.
(c) The WHOQOL-abbreviated questionnaire is used to measure the perception of quality of life. The questionnaire consists of 26 questions, with question 1 related to the perception of quality of life and question 2 related to satisfaction with health, and the average of these two questions assumes the self-assessment of quality of life. The remaining 24 facets make up 4 domains which are physical, psychological, social relations, and environment. The answers for each domain and the first two questions follow a Likert scale from 1 to 5, and the higher the score, the better the perception of quality of life. For its measurement, it is necessary to recode the value of the answers to questions 3, 4, and 26, as shown in the parentheses (1 = 5) (2 = 4) (3 = 3) (4 = 2) (5 = 1). All results are expressed as an average both in the domain and in the first two facets, as follows:

Self-assessment of quality of life (Q1 + Q2)/2
Domain 1—Physical (Q3 + Q4 + Q10 + Q15 + Q16 + Q17 + Q18)/7
Domain 2—Psychological (Q5 + Q6 + Q7 + Q11+ Q19 + Q26)/6
Domain 3—Social relationships (Q20 + Q21 + Q22)/3
Domain 4—Environment (Q8 + Q9 + Q1 + Q13 + Q14 + Q23 + Q24 + Q25)/8
*Q = Question.

After checking the averages above, the responses are classified by the following: needs improvement (when it is 1 to 2.9); regular (3 to 3.9); good (4 to 4.9), and very good (5).

### 2.4. Ethical Issues

This study was approved by the Human Research Ethics Committee (CEP) of the Tropical Medicine Center of the Federal University of Pará (UFPA), under opinion No. 5447240. When the participants were invited to participate in the research, they received a link, and when opened, they had access to the Free and Informed Consent Form (TCLE). It contained information about the research procedure and objectives of the work. After reading this term and before starting to fill in the research instruments, the participants had the option of clicking on the item “I have read the Free and Informed Consent Form (TCLE) and I agree to participate in the research”, otherwise, they were instructed to just click on “I disagree” and send the form.

### 2.5. Data Analysis

For the statistical analysis, the Statistical Package for the Social Sciences, version 24 was used, considering the significance level of *p* < 0.05. To compare attachment style and perceived quality of life between groups A and B, the Mann–Whitney test was used, due to the lack of normal data distribution, and Pearson’s chi-square test for categorical classification of perceived quality of life.

## 3. Results

A total of 100 adult mothers were evaluated, of which 45 mothers of children with DM1 were included in group A, and 55 mothers of children without chronic diseases were included in group B, with the mean age for both groups being 34.86 ± 6.2 years.

Most of the participants in group B had completed or incomplete higher education, which was statistically significant, where being the mother of a child without chronic diseases has a positive relationship with having higher education and a negative relationship with technical education, while in group A this relationship would be inverse, i.e., being a mother of a child with DM1 would have a positive relationship with technical education and a negative relationship with higher education.

It is possible to see in Table 1 that of the 100 participants, 55% were married and 33% were in a stable relationship. As for skin color, 56% said they were brown or black, while 41% said they were white. Most participants in group B were from the north of the country (about 60% of the sample); while the mothers in group A were mostly from the southeast region (about 24.44%). Regarding family income, 40% of the participants stated that their family income was between one and two minimum wages and 41% stated that their family income was between three and four minimum wages (a minimum wage: 1212 reais), with 78% of the mothers stated that they work outside the home. Regarding the classification of nutritional status according to BMI, there was no statistical difference between the classifications in both groups (Table 1).

Regarding the attachment style, 65% of the participants had an insecure attachment style, and of these, 47.6% were mothers of children diagnosed with DM1. Regarding the total number of participants who presented with characteristics of the insecure attachment style, 41.53% were classified with the anxious attachment factor, while 58.46% presented characteristics of the avoidant attachment factor. There was no statistically significant difference between the manifestation of the attachment style depending on the DM1 diagnosis (Table 2).

When comparing the groups of mothers of children with and without DM1, it was verified that there was a statistical significance between having low quality of life scores and being a mother of a child with DM1, when compared to the scores of mothers without chronic diseases. Concerning attachment factors, when comparing the anxiety and avoidance scores in the assessed groups, there was no significant difference (Table 2).

A correlation analysis was also performed between the attachment insecurity factors (anxiety and avoidance) of mothers with children with DM1 and the domains of the perceived quality of life. In this correlation, a relationship was found only between the anxiety factor and the domains of social relations (s = −0.343) and the environment (s = −0.300), and between the avoidance factor and the domain of social relations (s = −0.321). These few correlations were negative and of a low level of statistical significance (*p* = 0.01). That is, there is little relationship between the attachment factors (anxiety and avoidance) and perceived quality of life in mothers of children with DM1.

As for the perception of quality of life subdivided into domains, it was found that the mothers of children with DM1 had a statistically worse perception of their quality of life when compared to the perception of the mothers in group B in most of the evaluated domains (Table 3).

## 4. Discussion

The present study evaluated the relationship between adult attachment and the perception of the quality of life of mothers of children with DM1. The main findings in this research were the statistically significant relationship between being a mother of a child diagnosed with DM1 and a worse perception of the quality of life in the classifications of almost all evaluated domains. In addition, there was also a statistically significant relationship between the scores related to the worst perception of the quality of life and being a “pancreas mother” [6]. Brazil currently ranks third in terms of the global prevalence of DM1, with 51,500 children and adolescents aged 0 to 14 years [24]. According to the literature, being a mother of children with a related chronic issue generates a higher level of distress due to the care related to the disease [15,25,26].

When it comes specifically to the mothers of children with DM1, the feeling of having a child with this condition is linked to the end of the dream of having a healthy child, in addition to living with the fear of loss, as well as dealing with factors related to the disease, such as having to apply insulin, check blood glucose, treat hypo- and hyperglycemia crises [9,26,27,28], in addition to the social isolation of the family, often with the mother as the main caregiver, due to the reduction in their participation in festive gatherings due to the need for dietary adjustments, a factor that may predispose to the emergence of psychological disorders, such as depression [15,25,29,30,31].

In addition, in both evaluated groups (A and B), high percentages were verified in the “regular” and “needs improvement” classifications in the evaluated quality of life domains. This finding suggests that even if a mother does not have a child with a chronic disease, she may have a worse perception of quality of life, which is probably due to the accumulation of roles directed at women: remaining responsible for caring for and educating children, and in many cases also being responsible for household chores in addition to working outside the home (to help support the household and acquire financial independence) [32].

More than half of the Brazilian population is in social classes D and E, with a monthly income of two to four minimum wages [33]. In this research, more than 80% of those evaluated are within this range. According to the 2019 Household Information and Communication Technologies (ICT) survey, classes A, B, and C make up 80% of the Brazilian population with access to the internet, and that economic classes E and D, despite having internet access, use a limited number of data, and in many cases use internet only on cell phones, therefore, apparently, the purchasing power of this population influences and restricts access to the internet [34].

Another finding of this study concerns maternal education, in which being a mother of a child without chronic disease was positively related to having a complete higher education and negatively related to a technical qualification, and conversely, being a mother of a child with DM1 was positively related to having a technical qualification and negatively in having a higher qualification. Despite this finding, 64.44% of the evaluated mothers in group A answered that they had completed or incomplete higher education, in addition to the fact that 24.4% of those evaluated in this group declared that they had technical training. The literature points out that maternal education is a key point for the treatment of children with chronic diseases, due to the greater demand for medical care, in addition to being positively associated with a reduction in child malnutrition [18,25,31,32,35,36,37].

In this research, most of the volunteer mothers who had children without the diagnosis of DM1 lived in the northern region of Brazil, probably due to the researchers involved working in this region and the dissemination of the questionnaire among the public through their social networks. However, when it comes to the mothers of children diagnosed with DM1, the distribution of participants at the national level was homogeneous, with the majority of respondents from the southeastern region of the country (24.44%), probably due to the wide dissemination in social networks and specific social events in the region that address the issue of diabetes.

Regarding the socioeconomic profile, there was no statistically significant difference between the family income of both groups; moreover, there was also no statistically significant difference between the groups of mothers who worked outside the home. On this last point, this study differs from the literature [15,16,37], as the mothers of children with chronic diseases often leave their jobs and dedicate themselves exclusively to caring for their children. However, it should be noted that the matriarchs in this study (group A) probably continue to work outside the home due to the need to supplement their family income, since 42.2% of those evaluated had a family income in the range of one to two minimum wages. However, it is still worth noting that this study took place in a pandemic period, despite the softening of protective measures. Brazil went through and is still going through a difficult scenario, which could have contributed to these findings and consequently could have interfered with the average family income in this population [26].

As for marital status, it was found that in both groups, most of the evaluated mothers declared to be married or in a stable relationship, with no statistically significant difference between the groups. According to the literature, having a partner is a positive factor for adapting to issues related to the illness, since single-parent families have greater difficulties in adapting to the disease, have higher levels of stress, and have more problems related to glycemic control [17,18,19,20]. In addition, families in which both parents are present also show positive results in DM1 care [9,24,27].

One of the expected results of this research was that the mothers of children with DM1 who had high anxiety or avoidance scores had a worse perception of their quality of life compared to those mothers with low anxiety and avoidance scores, since the literature in the area defines that attachment behaviors are expressed in stressful situations [28], such as having a child with DM1 in infancy [15,22]. The literature states that the mothers of children with some chronic issue apparently have higher levels of anxiety than mothers of typical children, as in the study by Yildiz, Narsat, and Apaydin, in 2023 [37], which studied the presence of anxiety in the mothers of children with chronic constipation. In this study, it was found that the more episodes of constipation the child had, the more anxious the mother was.

Other studies have already evaluated the relationship between having a child with a chronic illness and attachment style, such as the study by Al-Yagon and collaborators (2020) in Israel, a study carried out with 100 children and their mothers. These children were fifth- and sixth-graders, aged between 11 and 12 years old, half of whom were diagnosed with attention deficit hyperactivity disorder (ADHD) and the other half were typically developing. This study used the Manchester Attachment Story Task (MCAST) instrument and the Experience in Close Relationships (ECR) to assess the mothers, as did this study, to verify child attachment. As a result, it was pointed out that the children with ADHD, like their mothers, had higher percentages of insecure attachments when compared to the population with typical development, thus finding possible relationships between attachment style and the context of chronic illness [38].

With regard to the mothers of children with ADHD, in addition to the higher incidence of insecure attachment, greater stress and anxiety were also observed (Al-Yagon et al., 2017). Insecurity and overload often lead to overprotection and inadequate control, making it difficult to develop a safe base for the child, interfering with the ability to deal with therapeutic approaches directed at the child, which may hinder the indicated treatment. These depressive and anxious symptoms may be linked to maternal attachment insecurity [38].

In this research, however, no statistically significant difference was found between the attachment factors and the presence of the DM1 diagnosis with the perception of a worse quality of life. This may have happened due to the shared stress of motherhood. In addition, the life history of the participants could modify this relationship, as well as a possible psychotherapeutic follow-up, which was not observed in this study and should be considered in future studies.

This research presents unprecedented and relevant data but has some limitations, such as not having been carried out with a representative sample of the population of mothers of children with and without a diagnosis of DM1 in Brazil, despite having data from mothers of children with DM1 from all Brazilian regions. In addition, searches in the remote format can exclude people who do not have good access to the internet, in addition to limiting individuals who are not familiar with remote search tools.

Despite these limitations, this research had a national sample and presented results on the perception of the quality of life of Brazilian mothers, comparing profiles according to having or not having a child with DM1. In this sense, the present study can help the health team to look sympathetically at the caregiver, understanding that the well-being of the dyad (patient and caregiver) directly influences adherence to treatment and the patient’s prognosis, since this illness is familiar [6,8]. Future studies are suggested with a representative sample from all Brazilian regions to verify the relationship between the caregiver’s quality of life and minor clinical complications for children with DM1, as well as to test intervention strategies in this population.

## 5. Conclusions

According to the results presented, it is concluded that the mothers of children with DM1 have a worse perception of their quality of life compared to the mothers of children without chronic disease, mainly taking into account the physical, psychological, and social relationships. In this sense, the inclusion of care in the health of this population should be considered, through different interventions with the multidisciplinary team. This care may reduce the impacts caused by the perception of a worse quality of life of “pancreas mothers” and establish psychological interventions that strengthen the mental health of the public, with individual and collective actions and services.

## Figures and Tables

**Table 1:** Sociodemographic and economic data and classification of the nutritional status of Brazilian mothers of children with and without the diagnosis of type 1 diabetes mellitus residing in Brazil.

		Mothers of Children with DM1 Group A (n = 45)	Mothers of Children without DM1 Group B (n = 55)	*p*-Value *
		n (%)	n (%)	
Education	Incomplete high school	0 (0)	1 (1,8)	0.023 *
	Complete high school	5 (11,1)	6 (10,9)	
	Technical education	11 (24,4) (+)	3 (5,5) (−)	
	Incomplete higher education	7 (15,6)	4 (7,3)	
	Complete higher education	22 (48,9) (−)	41 (74,5) (+)	
Region of Brazil	North	9 (20)	33 (60)	0.160
	Northeast	9 (20)	5 (9,09)	
	Midwest	11 (24,44)	3 (5,45)	
	Southeast	9 (20)	7 (12,72)	
	South	7 (15,55)	7 (12,72)	
Marital status	Married	29 (64,4)	26 (47,3)	0.158
	Stable union	13 (28,9)	20 (36,4)	
	Single	3 (6,7)	9 (16,4)	
Work outside the home	Yes	32 (71,1)	46 (83,6)	0.133
	No	13 (28,9)	9 (16,4)	
Average family income	Between 1 and 2 salaries	19 (42,2)	21 (38,2)	0.114
	Between 3 and 4 salaries	22 (48,9)	19 (34,5)	
	Between 5 and 6 salaries	2 (4,4)	10 (18,2)	
	Between 7 and 8 salaries	2 (4,4)	5 (9,1)	
Health Service	Public Health System (Sus)	10 (22,2)	12 (21,8)	0.598
	Private	3 (6,7)	7 (12,7)	
	Public Health System (Sus) + Private	32 (71,1)	36 (65,5)	
BMI ^1^ classification	Malnutrition	1 (2,2)	0 (0)	0.485
	Eutrophy	24 (53,3)	29 (52,7)	
	Overweight	15 (33,33)	17 (30,9)	
	Obesity	5 (11,11)	9 (16,36)	

* Pearson’s chi-square test, considering *p* < 0.05; (+) = positive association; (−) = negative association, considering the analysis of adjusted residuals (1.95 or −1.95). ^1^ Body mass index.

**Table 2:** Comparison of nutritional status, quality of life, and attachment style between mothers with children with type 1 diabetes and those without type 1 diabetes living in Brazil, 2022.

Nutritional Status	Mother with Children DM1 (n = 45) Group A		Mother without Children DM1 (n = 55) Group B		*p*-Value *
	Average (±DP)	Median (P5–P95)	Average (±DP)	Median (P5–P95)	
**Body Mass Index**	25.28 (±3.69)	24.91 (20.70–31.77)	25.82 (±4.11)	24.91 (20.37–34.54)	0.675
**Quality of Life**	**Average (±DP)**	**Median (P5–P95)**	**Average (±DP)**	**Median (P5–P95)**	*p*-value *
Physical domain	3.15 (±0.53)	3.14 (2.18–4.14)	3.58 (±0.55)	3.71 (2.42–4.34)	<0.0001
Psychological domain	3.14 (±0.54)	3.16 (2.10–4.00)	3.44 (±0.64)	3.33 (2.00–4.53)	0.008
Social relationships	3.05 (±0.52)	3.00 (2.00–4.23)	3.36 (±0.68)	3.33 (2.00–4.06)	0.004
Environment	3.06 (±0.43)	3.00 (2.50–4.08)	3.30 (±0.52)	3.25 (2.47–4.27)	0.012
Self-evaluation	3.16 (±0.77)	0.60 (1.6–4.85)	3.38 (±0.75)	3.50 (2.00–4.50)	0.116
**Attachment style**	**Average (±DP)**	**Median (P5–P95)**	**Average (±DP)**	**Median (P5–P95)**	*p*-value *
Anxiety	3.78 (±1.39)	4.2 (1.12–5.68)	3.66 (±1.25)	3.80 (1.72–5.80)	0.534
Avoidance	2.71 (±1.00)	2.60 (1.00–0.03)	2.37 (±0.78)	2.20 (1.16–4.00)	0.077

* Mann–Whitney test.

**Table 3:** Classification of the perception of quality of life of evaluated mothers of children with and without the diagnosis of type 1 diabetes mellitus residing in Brazil.

		Mothers of Children with DM1 Group A (n = 45)		Mothers of Children without DM1 Group B (n = 55)		*p*-Value *
		n	%	n	%	
Physical domain	Very good	0	0	0	0	0.003
	Good	3	6,7 (−)	19	34,5 (+)	
	Regular	31	68,9	29	52,7	
	Need to improve	11	24,24	7	12,7	
Psychological domain	Very good	0	0	0	0	0.043
	Good	5	11,1 (−)	16	29,1 (+)	
	Regular	27	60	31	56,4	
	Need to improve	13	28,9	8	14,5	
Domain of social relationships	Very good	0	0	1	1,8	0.041
	Good	3	6,7 (−)	15	27,3 (+)	
	Regular	31	68,9	28	50,9	
	Need to improve	11	24,4	11	20	
Domain of the environment	Very good	0	0	0	0	0.471
	Good	3	6,7	7	12,7	
	Regular	25	55,6	32	58,2	
	Need to improve	17	37,8	16	29,1	
Self-assessment of quality of life	Very good	2	4,4	1	1,8	0.020
	Good	6	13,3 (−)	21	38,2 (+)	
	Regular	29	64,4 (+)	21	3,8 (−)	
	Need to improve	8	17,8	12	21,8	

* Pearson’s chi-square test, considering *p* < 0.05; (+) = positive association; (−) = negative association, considering the analysis of adjusted residuals (1.95 or −1.95).

## Data Availability

The data is not publicly available as it contains the personal information of the participants involved. Therefore, the data of this work is confidential, to maintain the privacy of those involved.
